# Looking for a perfect match: multimodal combinations of Raman spectroscopy for biomedical applications

**DOI:** 10.1117/1.JBO.26.8.080601

**Published:** 2021-08-12

**Authors:** Iwan W. Schie, Clara Stiebing, Jürgen Popp

**Affiliations:** a Leibniz Institute of Photonic Technology, Jena, Germany; b University of Applied Sciences—Jena, Department for Medical Engineering and Biotechnology, Jena, Germany; c Friedrich Schiller University Jena, Institute of Physical Chemistry and Abbe Center of Photonics, Jena, Germany

**Keywords:** Raman spectroscopy, optical coherence tomography, fluorescence imaging, label-free, *in vivo*

## Abstract

Raman spectroscopy has shown very promising results in medical diagnostics by providing label-free and highly specific molecular information of pathological tissue *ex vivo* and *in vivo*. Nevertheless, the high specificity of Raman spectroscopy comes at a price, i.e., low acquisition rate, no direct access to depth information, and limited sampling areas. However, a similar case regarding advantages and disadvantages can also be made for other highly regarded optical modalities, such as optical coherence tomography, autofluorescence imaging and fluorescence spectroscopy, fluorescence lifetime microscopy, second-harmonic generation, and others. While in these modalities the acquisition speed is significantly higher, they have no or only limited molecular specificity and are only sensitive to a small group of molecules. It can be safely stated that a single modality provides only a limited view on a specific aspect of a biological specimen and cannot assess the entire complexity of a sample. To solve this issue, multimodal optical systems, which combine different optical modalities tailored to a particular need, become more and more common in translational research and will be indispensable diagnostic tools in clinical pathology in the near future. These systems can assess different and partially complementary aspects of a sample and provide a distinct set of independent biomarkers. Here, we want to give an overview on the development of multimodal systems that use RS in combination with other optical modalities to improve the diagnostic performance.

## 1 Prologue

In our previous publication, “*In-vivo* Raman spectroscopy: from basics to applications,”^1^ we have provided a comprehensive review on a variety of technological developments and *in vivo* applications for Raman spectroscopy (RS). The goal of the aforementioned review was to outline to novice researchers the important factors, which are crucial for the translation of RS to *in vivo* applications. We discussed instrument parameters that are most relevant and suitable for developing Raman systems in a clinical setting. We have also outlined a variety of possible fiber-probe implementations, which are readily available, and included various designs for endoscopic probes, e.g., ball-lens focusing and side-viewing probes. Also, handheld probes, which can be used for the characterization of molecular information on surfaces, were presented. Additionally, we briefly touched on spatially offset Raman spectroscopy (SORS) and implementations that include image guidance. The reviewed applications focused on cardiovascular diseases, inflammatory diseases, and cancer. The latter was separately reviewed for brain, skin, head and neck, breast, digestive and urinary system, and prostate and cervix. In essence, our previous review provided an in-depth assessment on instrumentation and *in vivo* applications of RS. A major topic that we touched only briefly in the previous review is the multimodal implementation of RS for *in vivo* diagnostics. However, we truly believe that in the near- and long-term future, the implementation of multimodal optical diagnostic systems will be one of the most important topics and drivers for a clinical translation, and RS will play an exceptional role in this translation. This review provides an overview on the different combinations of RS with other optical modalities.

## 2 Introduction

The principal tool for pathological analysis of tissue samples is white-light microscopy. The contrast of white-light microscopy is due to reflection or attenuation of the illumination light and generally results in low contrast and low signal specificity. To improve the specificity, tissue sections are commonly stained by hematoxylin and eosin (H&E), which allows the visualization of nuclei through the application of eosin and cytosol and the extracellular matrix (ECM) through the application of hematoxylin.^2^ The former provides a blue hue, whereas the latter provides a pink hue of the stained areas. This staining approach has withstood the test of time and, as of now, is the gold standard for disease diagnostic in clinical pathology.^3^ While decades ago, the sample preparation was mostly a manual and labor-intensive process, nowadays, due to extensive automation steps in digital pathology, scans can be digitized fast and in automated fashion, and extensive research efforts are going into automated data analysis and annotation procedures.^4^ Nevertheless, a major drawback of staining-based approaches is that despite the automation procedures, the diagnostic results are not immediately available, and patients have to wait for an extended period of time to receive results.^5^ This can be different when the analysis is required intraoperatively, such as in oncological resection surgery, where a pathologist analyses frozen sections parallel to the surgery to determine whether the removed tissue still contains tumor cells or whether the malignant tissue was entirely removed. However, there are also problems herein that the surgeon cannot assess or determine the grade of the tissue during the resection, as the information is not immediately available, and additionally, the histological grade based on frozen sections might even differ from the final diagnosis.^6^ New ways to establish resection margins *in vivo* or to determine the grade and stage of a tumor would be highly beneficial. In the last two decades, there has also been a significant push to improve and augment the imaging information of traditional microscopy and endoscopy using unique physical tissue properties other than the reflection and refraction of light.^7^

Fluorescence-based techniques are the go-to method for many biomedical applications, providing specific information based on extrinsically applied labels. The applications range from protein expression in single cells but also in entire small model animals,^8^^,^^9^ chromosome imaging,^10^ calcium imaging,^11^ fluorescence-activated cell sorting,^12^ and many more. Fluorescence *in vivo* imaging is a good example for the translation of an optical technology from the lab to clinical applications, where specific exogenic dyes are applied to improve the visualization of malignant tissue.^13^ Multiple organic dyes, which are approved by the U.S. Food and Drug Administration for human applications are readily available, including indocyanine green, 5-aminolevulinic acid, and methylene blue.^14^ Nevertheless, these dyes can lack specificity, as they stain the vasculature or the metabolic activity of cells, but do not provide any morphological or molecular specificity of the pathological tissue. Moreover, the medical approval of exogenic contrast agents is time consuming and a tedious process, making the development and translation of new and more specific staining approaches cumbersome. To improve on the specificity while removing the necessity for exogenic contrast agents, significant research effort has focused on the development of label-free, but molecularly and morphologically specific techniques that can overcome the above-mentioned complications while improving diagnostic results. Recently, a variety of these label-free methods, such as RS, coherent anti-Stokes Raman scattering (CARS) microscopy, stimulated Raman scattering (SRS) microscopy, second-harmonic generation (SHG), autofluorescence (AF) microscopy, including spectroscopy and fluorescence lifetime imaging (FLIM), and many others have shown very promising results for a label-free but molecular specific characterization of cells and tissue samples.^15^^–^^20^ Linear and non-linear RS for clinical applications have been extensively reviewed.^21^^–^^24^ While fluorescence-based techniques based on labels are most advanced in the clinical setting, FLIM is gaining more and more recognition as a multi-scale imaging technique^25^ and fundus imaging based on AF signals is becoming increasingly used in ophthalmology.^26^ To achieve a successful translation of molecular imaging techniques into the clinical environment, a major advantage can be the implementation into already routinely used medical devices, such as the da Vinci system (Intuitive Surgical, Sunnyvale, California). Pinto et al.^27^ designed a Raman endoscope, which can be grasped and positioned by a robotic arm, to improve minimally invasive surgery of the prostate. Various studies have focused on the implementation of molecular imaging techniques in robotic-assisted surgery and have been recently compiled in a review.^28^ Although many studies focus on single modalities, they have in common that each modality can only access a particular aspect of the sample’s property, potentially providing a lopsided view of the underlying pathology or with low diagnostic significance. Multimodal optical systems, which allow to extract complementary information of a sample based on different contrast mechanisms, could improve the diagnostic performance by assessing a larger set of biomarkers and pave the way for a broader translation of new optical tools for *ex vivo* and *in vivo* clinical diagnostics.^29^

In the following review, we present an overview of multimodal solutions for medical diagnostics from the perspective of RS. These systems allow to rapidly measure label-free molecular, morphological, and metabolic information. The targeted applications are tissue characterization of *ex vivo* samples with the future direction toward fiber-optical probe developments for *in vivo* applications. We describe different optical modalities and their advantages in multimodal systems, where we also provide an overview of recent research literature about their combination with RS.

## 3 Optical Modalities for Medical Diagnostics

In this section, we provide an outline of individual optical modalities including their benefits and drawbacks as well as their potential for multimodal applications. Table 1 summarizes the common modalities researched as diagnostic tools, with the emphasis on techniques readily combined with RS. These are morphologically sensitive optical coherence tomography (OCT), the fast and molecular-sensitive fluorescence-based techniques, and coherent RS applications exploiting non-linear optical effects.

**Table 1:** Summary of common optical modalities, which were readily combined with RS for tissue analysis.

Modality	Excitation wavelengths in nm	Signal wavelengths in nm	Resolution in μm	Contrast mechanism
RS	785	800 to 1060	Diffraction limited, but commonly a wider focus, e.g., 50 to 100 μm is used	Molecular bond vibrations
	1064	1087 to 1640		
OCT	800 to 1500	Identical to the illumination wavelength	Central wavelength and bandwidth limited, typically 5 to 10 μm	Ballistic scattering of photons from microstructures
AF and FLIM	360 to 560	380 to 700	Diffraction limited	Collagen, adenine dinucleotide, nicotinamide, porphyrins and flavin
FS	Visible – NIR, dependent on dye	Above excitation wavelength of chosen dye	Diffraction limited	Targeted molecules depending on chosen dye
SHG	800 to 1000	Half of the excitation wavelength	Diffraction limited	Non-centrosymmetric materials, e.g., collagen, myosin, tubulin
CRS	900 to 1100 (Stokes) 700 to 960 (pump) depends on the optical system and the vibrational band of interest	550 to 700	Diffraction limited	Molecular bond vibrations

### 3.1 Raman Spectroscopy

**Fig. 1:**
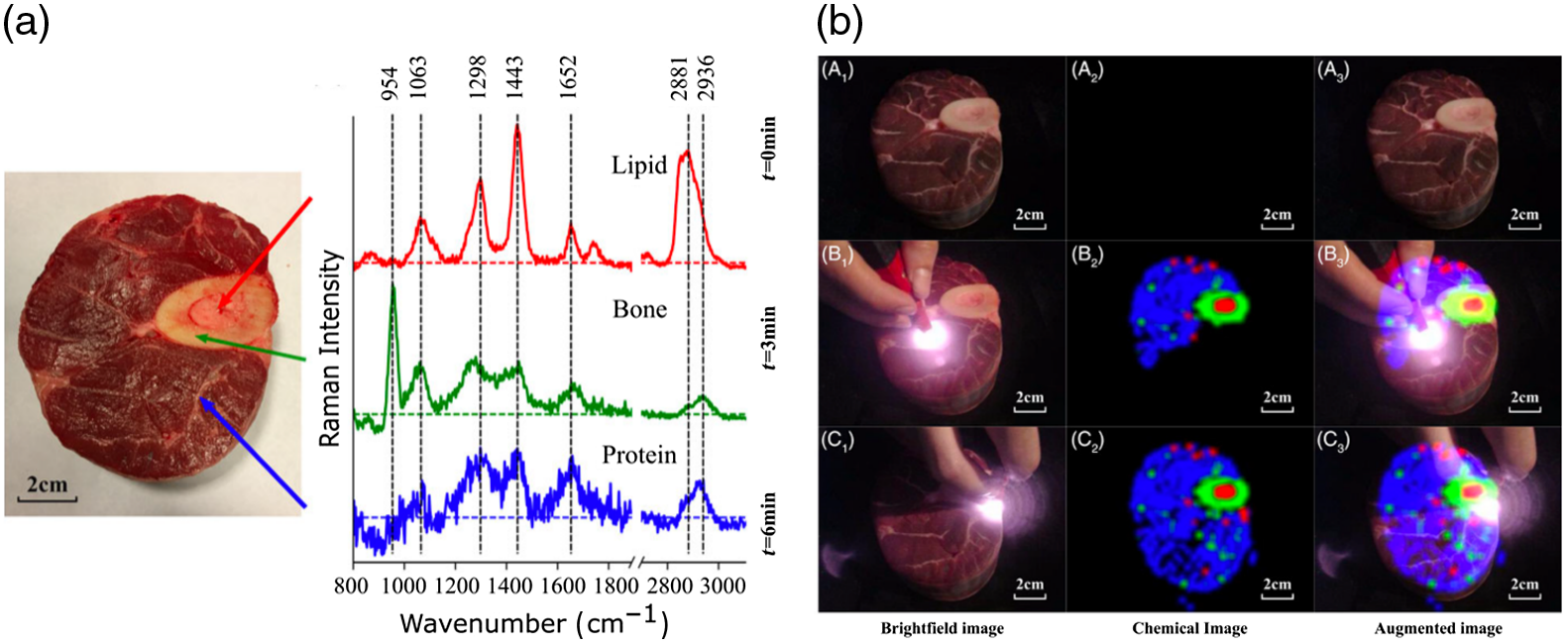
Previous implementations of fiber-based RS have been used for a point-based measurement of tissue, without any imaging information of the tissue of interest. Yang et al.^32^ have nicely demonstrated that a hand-held, fiber-based Raman probe can actually be used for imaging applications of biological tissue, which has significant potential for the detection of tumor margins. (a) Bright-field image with Raman spectra of indicated molecular signatures for lipid, bone and protein. (b) The implementation of a fiber-based scanning approach in combination with real-time data analysis and augmented chemical reality allows a direct visualization of the distribution of the molecular constituents in the sample. Here, row A displays the three different types of information, i.e., bright field, chemical image, and augmented image. Rows B and C display the information at two different timepoints, 3 and 6 min, respectively. The chemical and augmented image show a color-coded distribution of the three macromolecules from (a), i.e., red for lipid, green for bone, and blue for proteins.

The application of RS for *in vivo* diagnostics bears an exceptional potential for providing molecular specific disease information, ranging from cancer to cardiovascular diseases.^30^^,^^31^ Due to the intrinsic contrast mechanism of vibrational energy states of molecules through narrow-band laser excitation, there is no need to apply stains or complex sample preparation steps, offering an ideal platform to study different types of diseases and to provide a diagnostic evaluation. A generated Raman spectrum represents a linear combination of vibrational modes of the tissue of interest and can be considered as a unique molecular fingerprint of the probing volume. As we have outlined in our previous review, there are a variety of instrumentational approaches for *in vivo* endoscopy applications with RS, and the transition to clinical devices is steadily progressing.^1^ Nevertheless, despite the many advantages for clinical diagnostics, there are certain constrains that researchers using RS have to cope with. A prime example is the data acquisition speed. For pure samples present at high concentrations, such as dimethyl sulfoxide, polystyrene, N-acetyl-para-aminophenol, or lipids, Raman signals can be acquired nearly at kHz rates, enabling a rapid acquisition of molecular maps. For biological tissue samples, the situation is more nuanced and typically reported acquisition times range between 0.5 and 10 s.^23^ As such, imaging-based RS in patients has particular challenges, primarily due to the low quantum yield of the inelastic Raman scatters of tissue components. It is not entirely impossible to rapidly acquire Raman images of biological tissue as was recently demonstrated by Yang et al.,^32^ where a new development of a fiber-based Raman imaging approach was introduced, allowing rapid Raman-based tissue characterization. The presented ChemLighter approach enables a fiber-probe–based molecular imaging of the tissue with real-time data analysis, enabling the visualization of molecular information and detection of molecular tissue boundaries *ad hoc* as augmented chemical reality (Fig. 1).

In general, however, the typical *in vivo* applications of RS remain point-based acquisition of target tissue, due to the low signal yield and the resulting low acquisition speed. Speed, however, is not always the only relevant factor for diagnostics. As the name suggests, the key factor is the established diagnostic information that can be determined by the modality, and in this regard, RS can provide exceptional performance in comparison to other optical modalities, because the retrieved information differentiates even small changes in tissue composition. A non-negligible aspect is the interpretation of the observed changes and modifications, which are used for the diagnostic differentiation between tissue types and disease stages. Because the Raman signal is a linear combination of the underlying macromolecules, it is not only possible to determine the present macromolecules in the sample but also to precisely establish the underlying changes in the ratios between the components. The assignment of characteristic bands to specific functional groups of biomolecules also enables the evaluation of the chemical composition of the sample. Multivariate analysis techniques,^33^^–^^35^ such as principal component analysis (PCA), multivariate curve resolution, cluster analysis, partial least squares, non-negative least squares (NNLS), or vertex component analysis allow for the compositional assessment, differentiation, and classification of tissues based on Raman datasets, such as Raman maps.

### 3.2 Optical Coherence Tomography and the Combination with Raman Spectroscopy

OCT is a well-established method in ophthalmology, enabling the visualization of micrometer-resolved cross-sectional images from mm depths, providing diagnostical information of anatomical changes of retinal layers.^36^ The OCT contrast arises from changes in optical properties of the turbid tissue, when light is scattered at tissue interfaces and scatterers within the tissue, both inducing local changes in the index of refraction in a sample, whereas in a homogeneous tissue, the signal transmission function would be described by the Lambert–Beer law.^37^ The principle of OCT is based on low-coherence interferometry, where a broadband low-coherence illumination source, in combination with an interferometric detection scheme allow the detection of scattered photons from tissue and the determination of the scattering depth based on the time-of-flight difference between the photons in the reference arm and the sample arm. Nowadays, Fourier-domain OCT systems in form of spectral domain are most commonly used and are based on the measurement of a spectral interferogram, which contains the information on the depth profile of the sample. A variation to that approach is swept-source OCT, which uses wavelength tunable excitation source to generate the interferogram. With OCT typical penetration depths of 1 to 2 mm can be readily achieved in biological tissues. The advantage is evident, as not many other optical methods can provide label-free depth-resolved features with micrometer resolution at video rate. Because the retina is highly layered, there is a significant contrast for ophthalmological applications of OCT since the contrast information arrives from boundaries between optically heterogeneous media. In homogeneous media, little contrast can be established and only the attenuation coefficient can be deduced. Because in various pathological tissues, the macromolecular constituents are homogeneous, even under changing physiological conditions, a measured OCT signal often results in low contrast and low specificity. Moreover, while OCT can rapidly provide structural information, it cannot determine molecular or metabolic information of the investigated tissue and relies on the expertise of the user and a time-consuming correlation to conventional histological thin section staining methods, such as H&E-staining. This is cumbersome in *ex vivo* settings and becomes even more challenging for *in vivo* applications, where the correlation between measurement locations and an extracted sample are not easily achievable. Consequently, a combination with a highly specific optical modality, such as RS, can be very beneficial, as OCT can rapidly provide depth-resolved morphological information, whereas the slower, but more specific RS, can provide the underlying information on the molecular composition of a tissue. Both modalities have been readily implemented in endoscopes and an endoscopic combination where both modalities are integrated bears significant potential for *in vivo* clinical diagnostics.^38^ For example, endoscopy-based OCT applications have readily been shown for urinary tract, brain, cardiovascular, and gastrointestinal applications,^39^^,^^40^ whereas RS has been applied *in vivo* to most organs, including the organs mentioned for OCT.^1^ To bridge the disadvantage of both modalities and to create a whole of two halves, in the last decade, there has been significant interest in the combination of OCT with RS for clinical diagnostics.

**Fig. 2:**
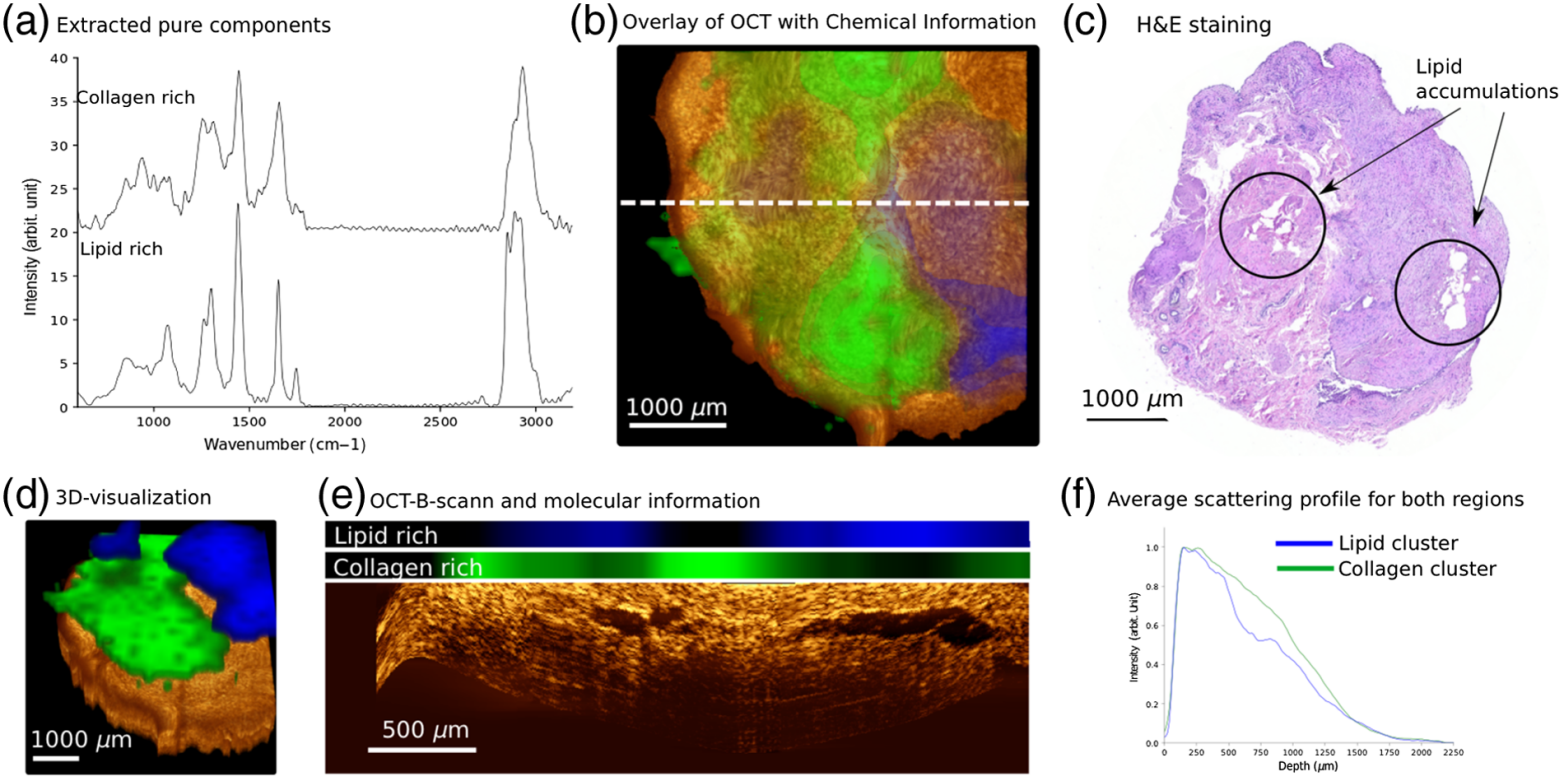
Combined imaging data using RS and OCT for a bladder biopsy is presented.^38^ (a) Raman spectra extracted from the data set reveal signatures of collagen and esterified lipids. These spectra were used to fit all Raman imaging data, using NNLS fitting. (b) The resulting coefficients were plotted on top of an OCT *en face* image and show the distribution of collagen and lipids in the biopsy. (c) A corresponding H&E slice from the same biopsy, indicates regions with potential lipid pools. (d) A 3D visualization of the OCT stack and the augmented molecular information. (e) B-scan from the biopsy indicates regions with an enhanced scattering and large voids. The Raman information is plotted above the B-scan and corresponds precisely to the identical location. It can be seen that the strong scattering regions correspond to collagen-rich regions, whereas the voids correspond to lipid-rich accumulations, clearly indicating the potential of the multimodal combination of RS with OCT. (f) Two A-scan, which were extracted using the Raman-maps of collagen and lipid as masks. As can be seen, the collagen-related A-scans have a higher scattering, whereas the lipid-related A-scans display a dip in the profile.

One of the first to demonstrate the advantage of a combined analysis using RS and OCT was Ko et al.,^41^ where both modalities were applied for the characterization of dental caries, using two separate optical systems, deducing that OCT could determine increased scattering from lesions and RS indicated changes in hydroxyapatite. The application of two separate optical setups has significant disadvantages, as it is quite challenging to precisely correlate the exact positions on the micrometer scale, and a combination of both modalities on the same system is desirable. Patil et al.^42^ presented such a combined RS/OCT system, which allowed a sequential acquisition with both modalities. The performance of the presented system was demonstrated on *ex vivo* breast cancer samples, where distinct features in OCT B-scans could be identified as protein and lipid-rich regions by RS. Furthermore, the system was also applied for an *in vivo* sampling of scab and a peripheral wound, where OCT contrast changes could be correlated to differences in collagen content. In a follow-up publication, the group also presented a portable instrument for the characterization of skin cancer.^43^ The results clearly outline the advantage of a multimodal combination for RS, which provides biochemical information, with the less specific but rapidly acquired OCT information. Ashok et al.^44^ have shown that the combination of both modalities can actually help to improve the diagnostic outcome for the discrimination of colonic adenocarcinoma and normal colon. While OCT alone provided a sensitivity and specificity of 78% and 74%, respectively; and RS provides a sensitivity and specificity of 89% and 77%, respectively; the combination of both signals achieved 94% for both diagnostic values. The work used a combination of PCA on the Raman data and on texture analysis features of OCT data, followed by a support vector machine classification. An interesting publication by Rangaraju et al.^45^ investigated *ex vivo* burn wounds on porcine skin for the identification of the degree of the burn (superficial partial-thickness, deep partial-thickness, and full-thickness), showing that the combination of both modalities achieved an average accuracy of 85% for differentiating those wounds. The work indicates that the performance of OCT for classification is significantly worse than for RS. Other publications have also investigated the improvement in diagnostic evaluation, where morphological and molecular signatures of kidney, liver, and small intestines were studied with both modalities.^46^ Maher et al.^47^ showed on *ex vivo* tissue samples that the combination of OCT with confocal RS can be used to accurately determine depth-resolved, physiologically relevant concentrations of the microbicide drug Tenofovir, which is used to prevent the sexual transmission of HIV. A depth-sensitive Raman system, based on a confocal implementation, in combination with OCT was presented by Khan et al.,^48^ and validated on a layered phantom and resected mucosa tissue, showing a successful delineation of epithelium and stroma layers with both modalities. Another depth-resolved Raman approach was outlined by Chen et al.,^49^ where wavelength modulated SORS was combined with OCT, providing morphological information and Raman information from white brain matter at a depth of 0.6 mm. In two very interesting reports by Wang et al.,^50^^,^^51^ the development and application of a handheld combined RS/OCT probe was presented. The side-view probe with an length of ∼120  mm and a head size of 13  mm×8  mm, made it suitable for *in vivo* applications in accessible body orifices, such as the oral cavity. The *in vivo* application of the probe at different locations within the oral cavity has shown that some structures observable in the OCT B-scans have distinct molecular signatures. Atherosclerotic plaques were also studied with a combination of RS/OCT on a microscopic setup.^52^ Placzek et al. have recently presented an optical setup that combines two forward-viewing endoscopic probes (Raman and OCT-probes) for the *ex vivo* characterization of bladder biopsies.^38^ Because both probes were precisely aligned to each other, it was possible to acquire Raman and OCT images from identical locations. The Raman information was used to differentiate tumor and non-tumor regions with a sensitivity and specificity of 92% and 95%, respectively; and additionally, the grade of the tumor with a sensitivity and specificity of 77% and 81%, respectively. OCT, on the other hand, was used to determine the stage of the bladder tumor and achieved a sensitivity and specificity of 73% and 78%, respectively. In a follow-up publication, Schie et al. used the data to correlate the signals of the morphological and molecular information, to provide a better understanding on the interrelation between both modalities.^29^ Because both were acquired in an imaging fashion it was possible to precisely overlay the imaging information of OCT and RS and deduce how signal features in OCT related to specific molecular components as shown in Fig. 2. It was shown that collagen, esterified lipid, and epithelium tissue could be very well correlated between the modalities, i.e., the scattering intensity will significantly differ between lipid and fibrous tissue, as well as their molecular composition for those tissues. The application of OCT in ophthalmology is quite established, but additional molecular information could significantly improve a variety of diagnostic parameters. Because the signal yield in RS is very low, for a long time, it appeared quite unfeasible to use RS for the investigation of the eye. Moreover, due to the presence of pigments, a strong AF signature would be expected. The first implementation, which attempted a combined RS/OCT sampling of the retina, was presented by Evans et al.^53^ The combined RS/OCT system could determine the molecular signature of a phantom sample, though the spectroscopic results for human and porcine retina samples were more challenging to interpret. In a recent publication by Stiebing et al.,^54^ it was shown that it is indeed possible to acquire interpretable Raman spectra from human retina samples even under the maximum permittable exposure limit. The results showed that a variety of macromolecular signatures, such as lipid, carotenoids, proteins, and nucleic acids could be extracted. Additionally, measurements on the same sample were also performed in combination with OCT.

Based on the presented evaluation, it is apparent that specifically the combination of RS with OCT is very powerful for the characterization of pathological tissue. Because classical RS implementations cannot rapidly provide depth information of the component distribution, the combination with OCT can add this by providing visual information of layered structures of samples and indicate transition zones of chemically different composed tissue locations, due to the change in the refractive index between the layers. For OCT, on the other hand, it is paramount to compare data of biopsies with pathological H&E-stained thin sections to establish ground truth information, which can be quite challenging for *ex vivo* samples and even more so for *in vivo* measurements. The combination of RS and OCT will provide significant benefits and enable the development of stand-alone diagnostic tools, which can provide rapid morphological and molecular information, without the need for additional pathological information.

### 3.3 Combination of Fluorescence-Based Approaches with Raman Spectroscopy

Fluorescence spectroscopy (FS) techniques are the most wide-spread techniques in biological and biomedical laboratories. Equipped with specific fluorescent tags or solely based on intrinsic AF, cellular or tissue composition and metabolic functions are routinely investigated, and even *in vivo* applications for image-guided surgeries are nowadays performed. For RS, tissue AF comes with a bad taste, as the endogenic fluorophores can be excited and thereby emit in the same wavelength range as the Raman signals but with several orders of magnitude larger intensities. This leads to a broad and intense background and consequentially shot noise, easily masking small Raman bands. To correct for AF background in Raman spectra, there is a multitude of computational and instrumental techniques.^55^^–^^60^ Nevertheless, the AF signal also carries valuable information about a sample, namely, its endogenous fluorophores, which could add beneficial information to improve classification rather than removing it by means of sophisticated chemometric algorithms.^61^^–^^64^ Obviously, a trade-off between a sufficiently strong Raman signal and manageable AF signal is still of utmost importance. Here, the excitation wavelength has a crucial impact and the AF background can be reduced by correctly choosing the excitation wavelength.^65^ While most biological materials exhibit their maximum absorption in the lower visible range around 500 nm, the Raman excitation wavelength can be chosen toward the NIR region of 785 nm or higher, where a lower fluorescence absorption cross section is present. This, however, also comes with a drawback of lower Raman signal as the intensity is proportional to $\lambda^{-4}$. A multimodal approach with one channel optimized for RS and one channel for optimal fluorescence detection can be an option. This concept goes back over a decade with studies evolving around the combination of AF imaging and RS, e.g., on gastric,^66^ skin,^67^ and breast^68^ cancer or during bronchoscopy.^69^ Kong et al.^70^ nicely demonstrated the benefit of combining different modalities, namely, a highly specific modality, such as Raman with a high-speed modality, such as AF imaging. An automated segmentation of AF images of tissue sections was implemented to select sampling points for RS, whose results were fed into spectral classification models for the diagnosis of basal cell carcinoma. They achieved an objective, label-free and fast classification in 20 to 60 min, which cannot be achieved with conventional histopathology. The method was optimized for and validated on a large-scale study on excised breast cancer tissue for the assessment of tumor margins during surgery.^71^ On an intra-operative timescale of only 12 to 24 min per specimen, the multimodal imaging technique was able to identify small residual tumors on the surface of breast excision specimens. Lin et al.^72^ developed an endoscopic system combining a total of four optical modalities to investigate *in vivo* nasopharyngeal tissue. White light and AF imaging provided a fast overview of the tissue, which allowed for guiding Raman and diffuse reflectance spectroscopy to suspicious regions to record more in-depth molecular information.

AF is limited too and depending on the presence of endogenous fluorophores in the targeted sample and frequently leads to rather unspecific information. Depending on the question at hand, this can create a major obstacle. FS with customized tags is preferred, if specific organelles or molecules of interest are present. For instance, fluorescence imaging in combination with Hoechst 33342 dye can reveal the cell nuclei, whereas proteins are highlighted by the green fluorescent protein (GFP) dyes, and lipids by the Oil Red-O or BODIPY dyes. However, RS can add more comprehensive information on different types of lipids, metabolites, proteins, and nucleic acids without labeling. This was demonstrated in a study of a cancer and non-cancer colon cell line, where Raman imaging allowed for a detailed view into cellular compartments, and fluorescence imaging with Hoechst 33342 and Oil Red-O was limited to the visualization of the nucleus and lipid-rich regions.^73^ However, the authors also demonstrated the strength of combining both modalities in an imaging fashion of identical locations in human colon tissues and cell lines, which allowed differences to be revealed in concentration and aggregation of a photosensitizer in cancerous and healthy sides. The added fluorescent photosensitizer did not disturb the Raman spectra, as the fluorescence was outside of the Raman wavenumber range. Unpublished results from our group have also shown that GFP dyes do not provide a strong background signal, when using 785 nm for the Raman excitation. Further studies combining fluorescence tags and RS include bladder cancer cells expressing the widely used GFP to monitor the distribution of proteins in living cells^74^ and the investigation of biochemical features of immune cells eosinophils and neutrophils.^75^ In addition to the here reviewed spontaneous RS, FS has also been combined in several studies with surface-enhanced Raman scattering using nanoparticles to enhance the singal.^76^^–^^79^

A rapidly evolving research area is the application of Raman tags and development of Raman probes.^80^ Raman tags can be stable isotopes, such as deuterium or C13-atoms, or functional groups, such as alkyne and nitrile, which create signals in a typically silent region of biological Raman spectra. A huge benefit is the relatively small size of the tags compared to the often bulky and large fluorescence dyes, which most likely alter the biological activity especially when introduced into cells. Gala de Pablo et al.^81^ circumvented the issue of bulky labels by designing a small photosensitizer, which is both a fluorescent and a Raman tag. Next to solvatochromatic fluorescence properties, two alkyne groups are building the Raman tag. Upon radiation with ultraviolet light, the photosensitizer can stimulate the production of reactive oxygen species causing cell death, hence, allowing the investigation of cellular behavior and biological activity. Li et al.^82^ synthesized a mitochondria-labeling tag for fluorescence and SRS imaging, which they demonstrated on living HeLa cells. The applicability of multiplexing a tag with additional EdU-labeling of the nucleus was also shown. Especially, the combination of fast coherent Raman and fluorescence techniques have a high potential for multiplexing using several Raman and fluorescence tags and thereby, breaking the often called color-barrier of stand-alone fluorescence.^83^^,^^84^

**Fig. 3:**
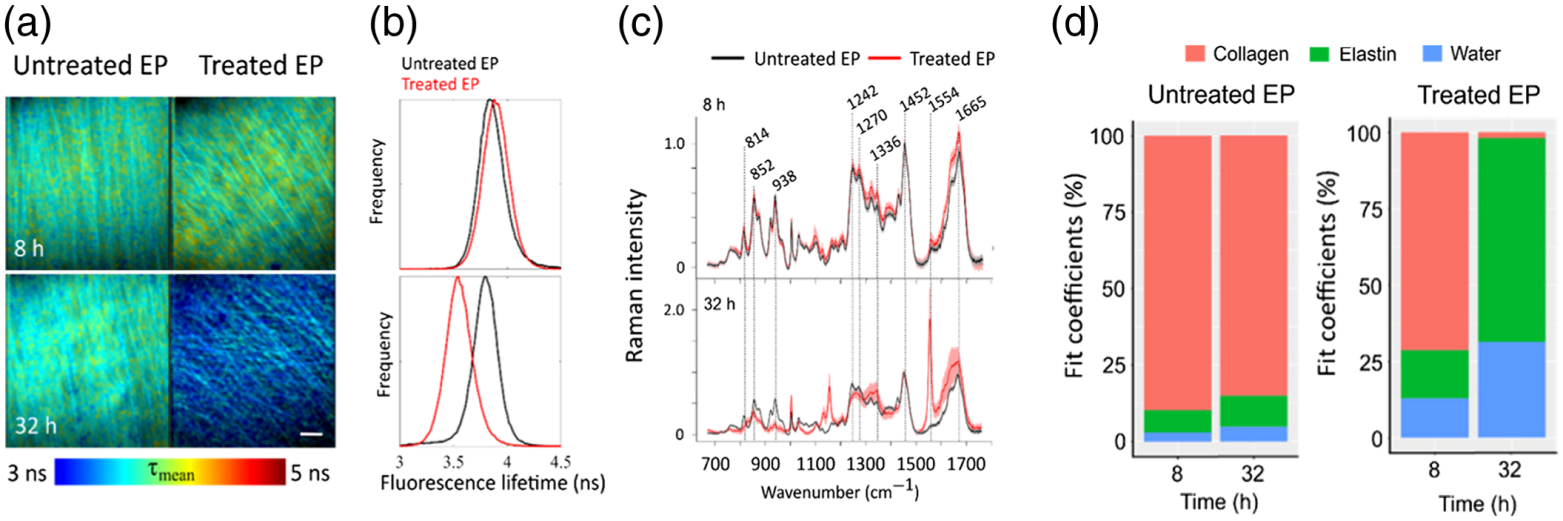
Multimodal study on decellularized equine pericardium samples treated with bacterial collagenase for two digestion time points of 8 and 32 h compared to untreated samples. (a) Fluorescence lifetime maps of untreated and treated samples display a distinct shift to lower lifetimes for the treated sample at 32 h. scale bar=20  μm. (b) A decrease in mean fluorescence lifetimes of all treated samples with the digestion time relative to untreated samples strengthens that statement. (c) Mean normalized Raman spectra of three untreated (black) and digested (red) samples uncover that the lifetime shift correlates with increased elastin bands in the treated samples at 32 h. (d) A non-negative least-squares fitting algorithm reveals the reduction of collagen and increase of elastin for digested samples over time. Reprinted under a Creative Commons Attribution (CC-BY) 4.0 international license from Shaik et al.^89^

In the past few years, studies of the combination of Raman with FLIM emerged but are still scarce.^85^^–^^87^ FLIM can monitor metabolic, molecular, and cellular functions, where the information arises from lifetimes decay properties of endogenous fluorophores. Additionally, contrast is generated by changes in the lifetime due to changes in the fluorophore’s microenvironment within the sample. The signal origin is therefore comparable with AF but can potentially result in better differentiation of endogenic molecules. Most common biomolecules, which exhibit a strong UV absorption, are a good target, and similarly to AF, include structural proteins, such as collagen, a dominant component of the ECM, and the structural protein elastin. These proteins are the backbone of connective tissue and can provide information on the tissue organization, which is often modified in pathological conditions. In addition to the proteins, lipid droplets can contain fluorescent molecules, such as retinoids and lipid oxidation pigments, which are associated with pathological processes. Furthermore, an important contrast mode are coenzymes, e.g., nicotinamide adenine dinucleotide phosphate and reduced flavin adenine dinucleotide, which are crucial in metabolic oxidative phosphorylation. Unfortunately, due to a high sensitivity, the interpretation of the lifetime values is quite complex, and a variety of factors can modulate the lifetime. Hence, the combination with RS can be quite beneficial, as it can precisely determine the molecular composition of the sample. Romero et al.^88^ studied the effectiveness of polymeric nanoparticles as potential doxorubicin drug delivery systems into cells using FLIM and Raman imaging. In a large-scale study, using four optical modalities SHG, two-photon fluorescence, FLIM, and RS, Shaik et al.^89^ investigated the chemical, structural, and functional alterations in collagenous tissue upon incubation with collagenase and correlated the results with mechanical strength revealed by atomic force microscopy measurements. Figure 3 shows the time-dependent digestion of decellularized equine pericardium by bacterial collagenase with a clear shift toward lower fluorescence lifetimes and a distinct change in the spectral profile of corresponding Raman spectra. FLIM and RS have previously also been combined in a fiber-based approach. Lagarto et al.^87^ implemented FLIM by means of time-correlated single-photon counting in the visible range with RS in the NIR. Dochow et al.^90^ also used a bimodal probe, which was validated in an *in vivo* setting in mouse brain. FLIM allowed for a rapid characterization of the biochemical features of the tissue, whereas Raman was guided specifically to regions of interest to provide high chemical specificity. In a follow-up publication, the same probe was used to investigate human atherosclerotic lesions.^91^ The fluorescence lifetime signals were recorded at three channels (390/40  nm dominated by collagen, 452/45  nm dominated by elastin and lipids, 542/50 nm dominated by extracellular lipids) after excitation with 355 nm, whereas 785 nm was used as a Raman excitation wavelength. Due to different tissue penetration depth of the wavelength, complementary information from the inner lipid-rich core and possible calcification was obtained by RS and surface information of the intima and the presence of fibrous caps was gathered by FLIM. An in-depth characterization of the origin of FLIM contrast in atherosclerotic lesions was investigated by Bec et al.^92^ using an adapted version of the before-mentioned bimodal probe. Only through the high specificity of RS, the increase in fluorescence lifetime in the violet spectral range (387/35  nm channel) in atherosclerotic lesions, which previously was linked to collagen, were actually associated with lipoprotein accumulation. This again demonstrates the benefit of combining the highly specific Raman modality with a high-speed fluorescence-based modality such as FLIM.

It becomes apparent that the combination of RS with fluorescence-based techniques yields great potential in accelerating the diagnostic evaluation process. The high-speed techniques AF and FLIM can overcome the intrinsically slow acquisition of Raman spectra and provide a quick overview of whole tissue sections and identify suspicious regions, which then can be analyzed in-depth by RS. Especially for tumor resection surgeries, this multimodal approach can offer a huge advantage in bedside diagnostics as laborious staining procedures can be circumvented. The strength in the combination of FS with RS lies in the development of tags active for both modalities, which open a pathway for targeted cancer treatment, as has been presented in the successful development of photosensitizers. As fluorescence techniques are already routinely used for medical diagnostics, the translation and acceptance of RS techniques in the clinical environment can greatly benefit from this combination.

### 3.4 Other Interesting Combinations

**Fig. 4:**
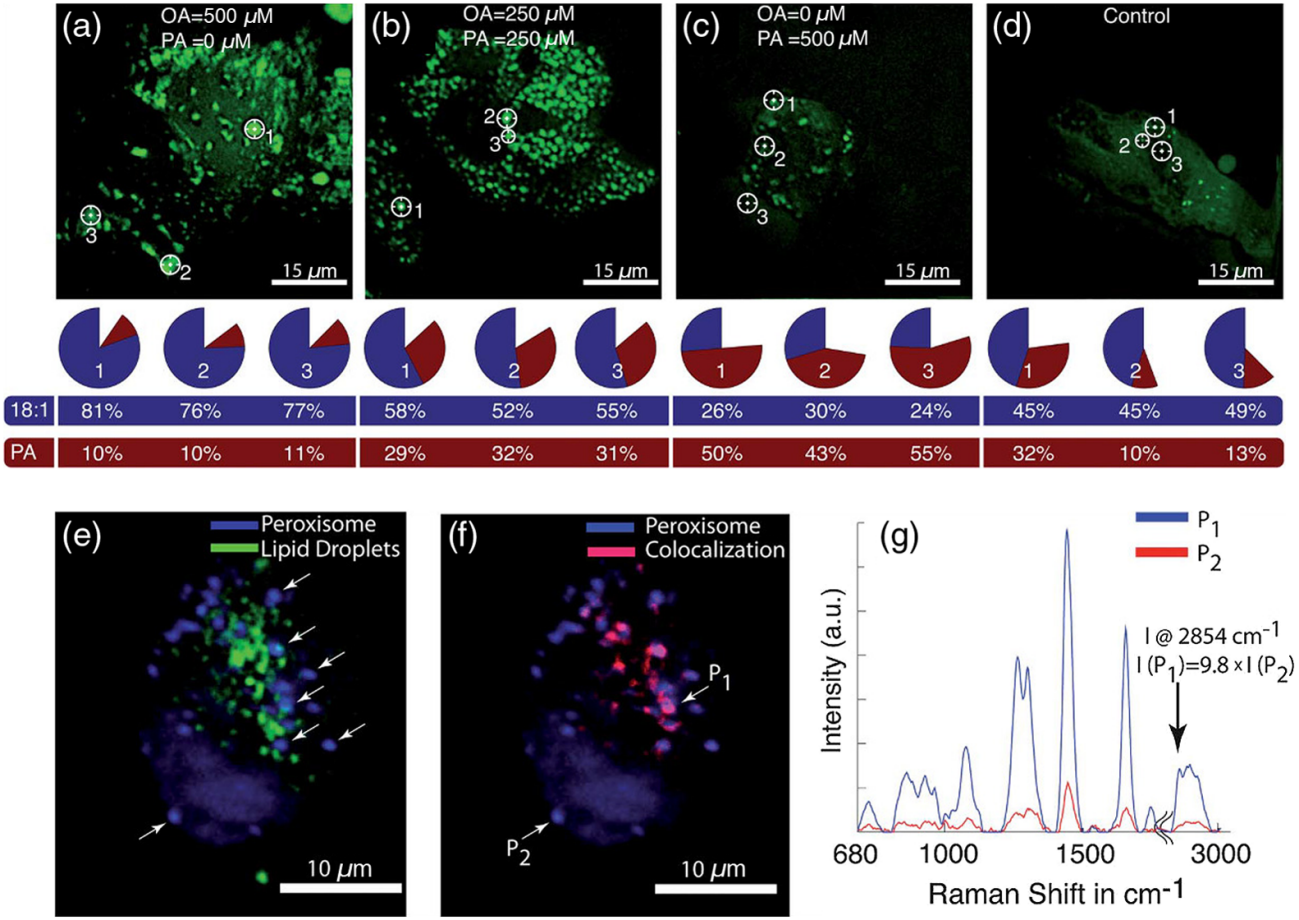
A combined system of CARS/TPEF system with RS, developed by Schie et al.^96^ applied to a label-free lipidomics on cells. (a)–(d) The intracellular distribution of lipid droplets exposed to different concentrations of two fatty acids, i.e., oleic acid and palmitic acid, and control was acquired by a CARS system. From indicated points, Raman spectra were acquired of the lipid droplets and the relative concentration for the two fatty acids calculated and plotted in a pie-chart, displayed below the images. Additionally, the gas chromatography values we established, showing superb correlation between those two methods. (e) Overlay of CARS and TPEF images on the same setup, showing cellular lipid droplets (green) and peroxisomes (blue), respectively. Raman spectra of peroxisome were taken from indicated locations. (f) The colocalization analysis between the TPEF image of peroxisome, and the CARS image of lipid droplets. The red regions indicate areas of high colocalization. (g) Based on the Raman signature from the two locations, P1 and P2, the difference in intensity between areas of the TPEF and the CARS image that are colocalized. The arrow indicates the ${\mathrm{CH}}_{2}$ stretch vibration $2854\;{\mathrm{cm}}^{-1}$ used for our CARS imaging, and the intensity difference between P1 and P2. Reproduced from Ref. 96 with permission from the Royal Society of Chemistry.

While OCT and fluorescence-based techniques are the most investigated combinations with RS, there are also other intriguing modalities, which could also benefit. One very surprising combination is with coherent Raman scattering (CRS) approaches, i.e., CARS and SRS microscopy. As the name suggests, both modalities are based on the Raman effect, however, in an enhanced way. CARS is based on a four-wave-mixing process and a coherent enhancement of the signal generation, where typically a single vibrational bond is probed, though tunable lasers are used to sample the entire high-wavenumber region (2800 to $3100\;{\mathrm{cm}}^{-1}$).^93^ There are also implementations for broadband CARS, which have shown quite interesting potential.^94^ Nevertheless, most implementations are performed in the high-wavenumber region, which contains only a limited number of vibrational bonds. Having a combined setup offers the opportunity to utilize the entire Raman region for molecular profiling. The first compound system using CARS and RS was presented by Slipchenko et al.^95^ who have used the system for a rapid acquisition of CARS images of cells, followed by subsequential acquisition of Raman spectra of intra-cellular lipid droplets to determine the endogenous and exogenous lipid content. Because CARS excitation sources also inherently generate SHG and two-photon excited fluorescence (TPEF), these systems are inherently multimodal. Schie et al. have previously described a compound CARS, SHG, and TPEF microscope, which had an additional RS unit. The system allowed to rapidly acquire large field of view label-free images of tissue samples and cells containing lipid droplets.^96^ In this study, we used CARS to identify regions of interest in cells, which were fed two types of fatty-acids and used the Raman unit to determine the fatty acid ratios in individual lipid droplets and compared the results to gas chromatography [Figs. 4(a)–4(d)]. We could show that it is possible to determine the fatty acid content on the single lipid droplet level. Moreover, we used the TPEF and CARS to visualize stained peroxisome and lipid droplet, respectively, and subsequently used RS to establish the molecular lipid profile of those components [Figs. 4(e)–4(g)]. Others have used the CRS/RS combination for the investigation of *Caenorhabditis* *elegans* nematodes,^97^ sinusoidal endothelial cells,^98^ hepatic microvasculature,^99^ and others.^100^^–^^102^ Klossek et al.^103^ have outlined the advantages of combining spontaneous RS with SRS for the investigation of lipid distributions in skin. The combination of both modalities helped to reduces the cross-sensitivity for blended Raman bands. In recent years, studies combining CRS with fluorescence techniques have emerged, such as the development of a FT-CARS/FT-TPE system as a high throughput flow cytometry system^104^ or the utilization of the stimulated Raman intermediate vibrational transition to excite into a higher fluorescent state.^83^ This shows that a combination of different Raman techniques but also CRS with fluorescence can have distinct advantages.

There are also other interesting optical modalities, which can be enhanced through the combination with RS, though much fewer reports are present. For instance, photoacoustic (PA) microscopy, which is based on the generation of an acoustic signal, using a light source while detecting the signal with an ultrasound transducer has been previously combined with RS. Probably one of the most groundbreaking and highly cited work on a combination was presented by Kircher et al.^105^ from the Gambhir group. Here, not only a combination of PA and RS was presented but also with magnetic resonance imaging, for the *in vivo* detection of glioblastoma in mice and a delineation of tumor margins *in vivo*. In other publications, the use of RS and PA was also outlined for vasculature imaging and characterization.^106^ Varkentin et al.^107^ presented a system that combines RS with PA and OCT and applied the system for the characterization of normal and melanocytic skin legions.

## 4 Conclusion

Conventional white-light microscopy lacks sufficient contrast, as it is only based on attenuation or reflection of light. To generate sufficient contrast in *ex vivo* biopsies the application of stains, e.g., H&E, is necessary to increase the specificity. Such staining approaches are not trivial to translate to *in vivo* studies and modalities that provide label-free and specific information are highly desirable. A variety of optical techniques which can assess different tissue-biomarkers have been investigated and recent research indicates that individual modalities have drawbacks and a combination of two or more modalities is highly desirable. In this review, we have provided an overview on multimodal combinations from the perspective of RS and outlined readily implemented combinations for an *ex* and *in vivo* characterization of a variety of diseases. The evaluation shows that RS was most commonly combined with fluorescence-based approaches and OCT. These combinations appear to have the most clinical and diagnostical potential, because the modalities complement each other well and compensate for the weakness of their counterparts. While both fluorescence imaging and OCT provide superb imaging speed in many applications, they can lack specificity, and the combination with the more specific modality, i.e., RS, is highly suitable. Moreover, there are two types of advantages which could be identified from using a multimodal combination: (1) improved diagnostic performance by combining the multimodal information, and (2) one modality can be partially used to explain features and observations of the other modality. Both aspects are very powerful, as the first clearly leads to better translation of the methods to the clinic and provides improved diagnostic information, whereas the second can provide valuable information about the sample and help to reduce the reliance on traditional staining approaches. The latter aspect will be specifically important for data interpretation of *in vivo* studies, where it is particularly challenging to correlate information with extracted biopsy samples. In addition to the two mentioned methods, combinations with CRS and PA microscopy could also have important implications, specifically to improve the diagnostic performance. The combination of the discussed optical modalities can add significant value for medical diagnostics and improve the speed with which the diagnostic results can be available. Especially, real-time diagnostics during surgeries with depth-resolved features in combination with molecular information can have lasting impact. As most of the techniques are label-free, cytotoxicity or metabolic changes can be circumvented. The decision on which modalities are suited best for a multimodal combination needs to be evaluated for each specific question at hand as it strongly depends on the underlying disease pathology and the associated changes in the tissue. RS has been readily applied to a large number of diseases providing their intrinsic molecular signature with very promising diagnostic results.^1^ The complementary modalities would ideally provide rapid imaging information to identify a region of interest where a Raman measurement can take place and/or provide additional biomarker information, e.g., morphological or metabolic. During the study design phase, it is important to understand what information will be provided by the individual modality and how well it describes the underlying pathological changes in the tissue. In addition to the assessment of the specific biomarker, the technological feasibility is also important since the requirements for the individual modalities do vary significantly. Starting from the excitation and signal wavelengths, which could span a range of hundreds of nm, the optical components, e.g., filters, coatings, or type of lenses, can generate background contributions. There are also multifold challenges for the *in vivo* translation, because complex systems have to be integrated into scanning fiber-optical probes, where the miniaturization and the excitation light delivery puts extreme constraints on the system.^38^^,^^39^ Nevertheless, technological developments of a variety of probe designs have been steadily progressing and offer a great opportunity for future developments and translation. To summarize, the combination of multimodal systems offer new opportunities to improve the diagnostical value by assessing a large number of orthogonal biomarkers, which will ultimately lead to a better translation of optical modalities into the clinics.
